# Supplementary material for: Suppression of SHROOM1 Improves In Vitro and In Vivo Gene Integration by Promoting Homology-Directed Repair
Source: Int J Mol Sci. 2020 Aug 13;21(16):5821. doi: 10.3390/ijms21165821 (PMC7461567; doi:10.3390/ijms21165821)

Figure 5b

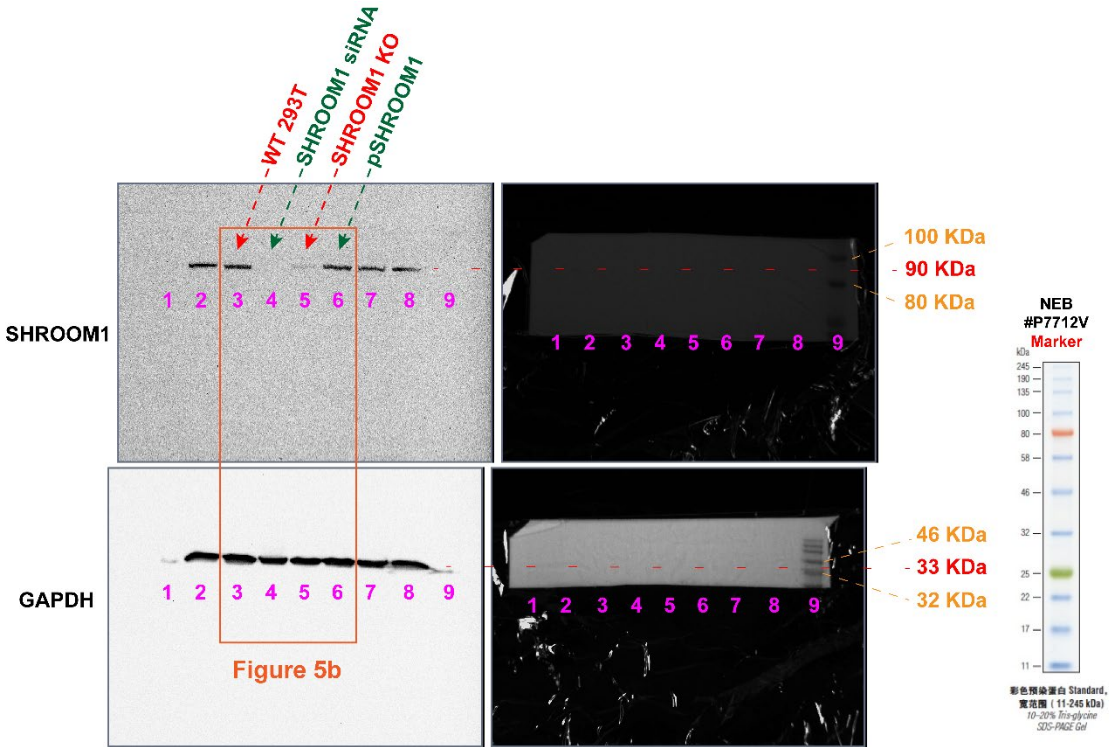

WT 293T
SHROOM1 siRNA
SHROOM1 KO
pSHROOM1
SHROOM1
GAPDH
1 2 3 4 5 6 7 8 9
100 KDa
90 KDa
80 KDa
46 KDa
33 KDa
32 KDa
Figure 5b
NEB
#P7712V
Marker
彩色预染蛋白 Standard，
宽范围（11-245 kDa）
10–20% Tris-glycine
SDS-PAGE Gel

Supplementary Figure S4a

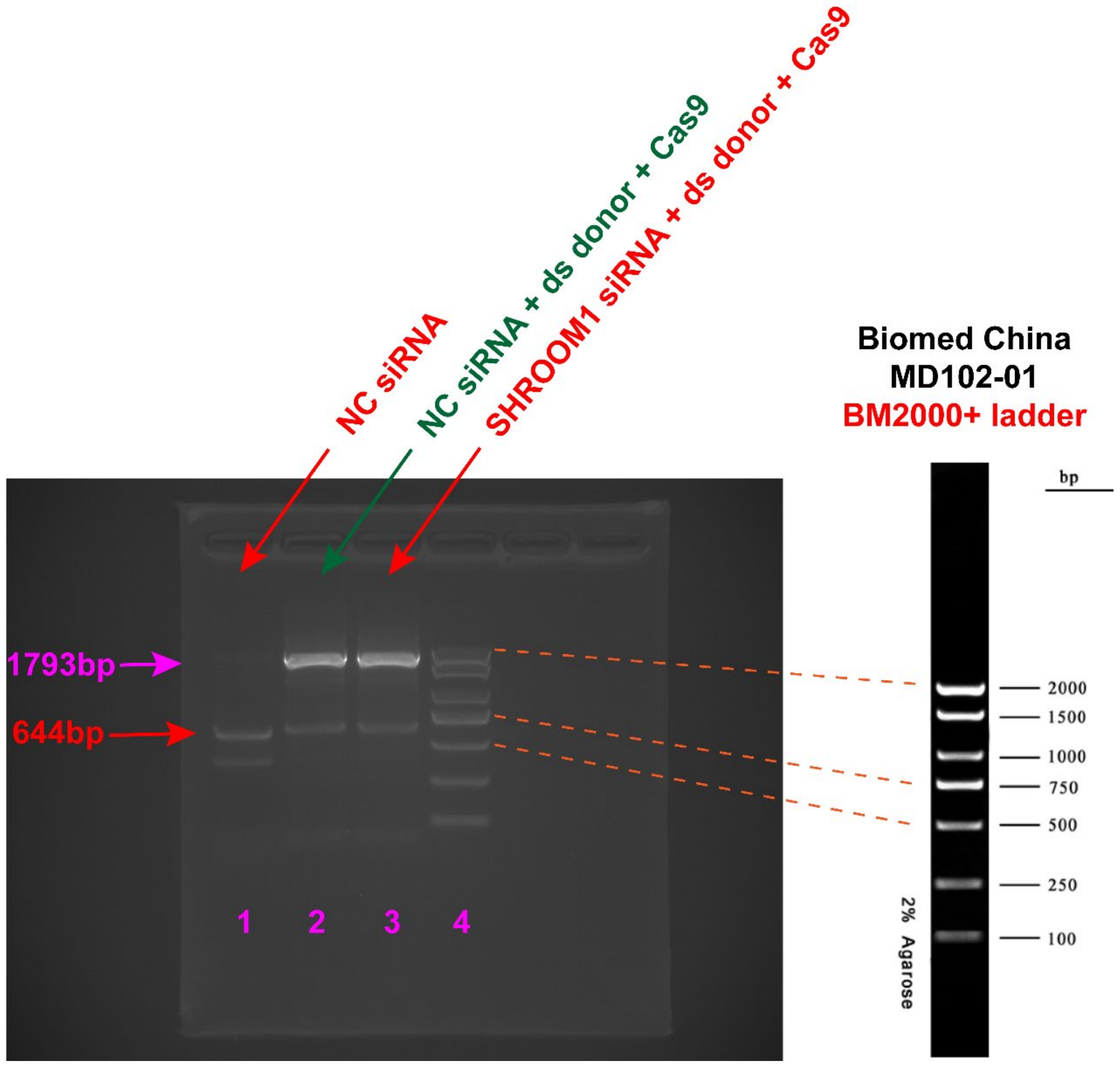

Supplementary Figure S4d

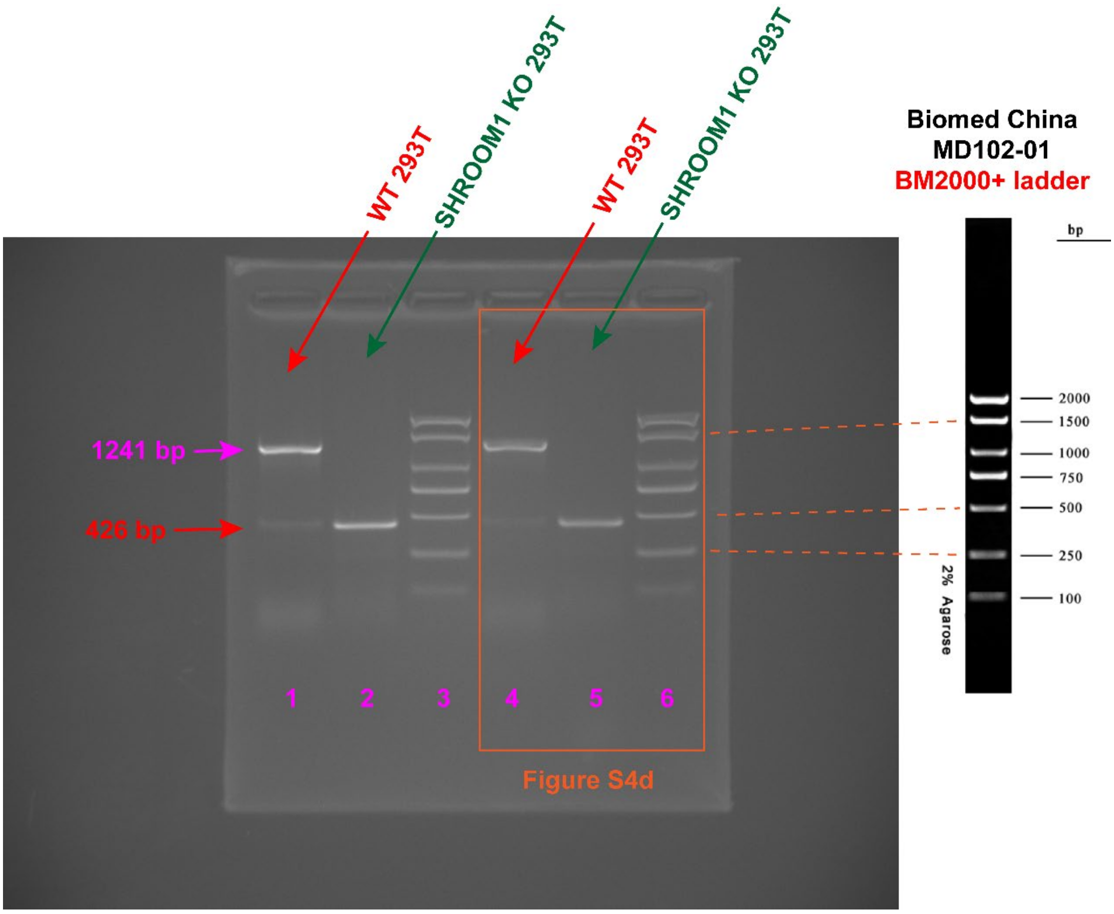

Supplementary Figure S5b

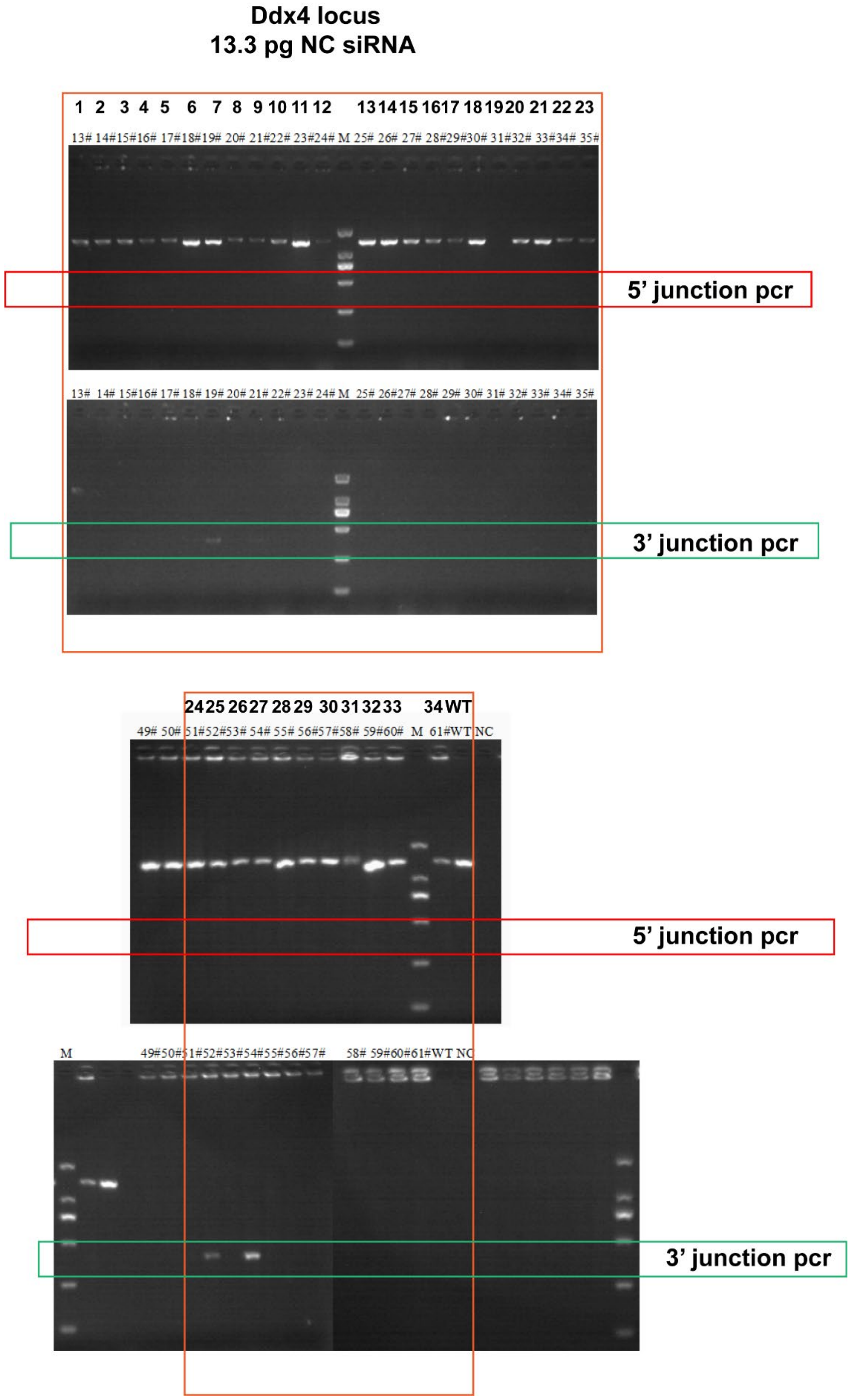

Supplementary Figure S5b

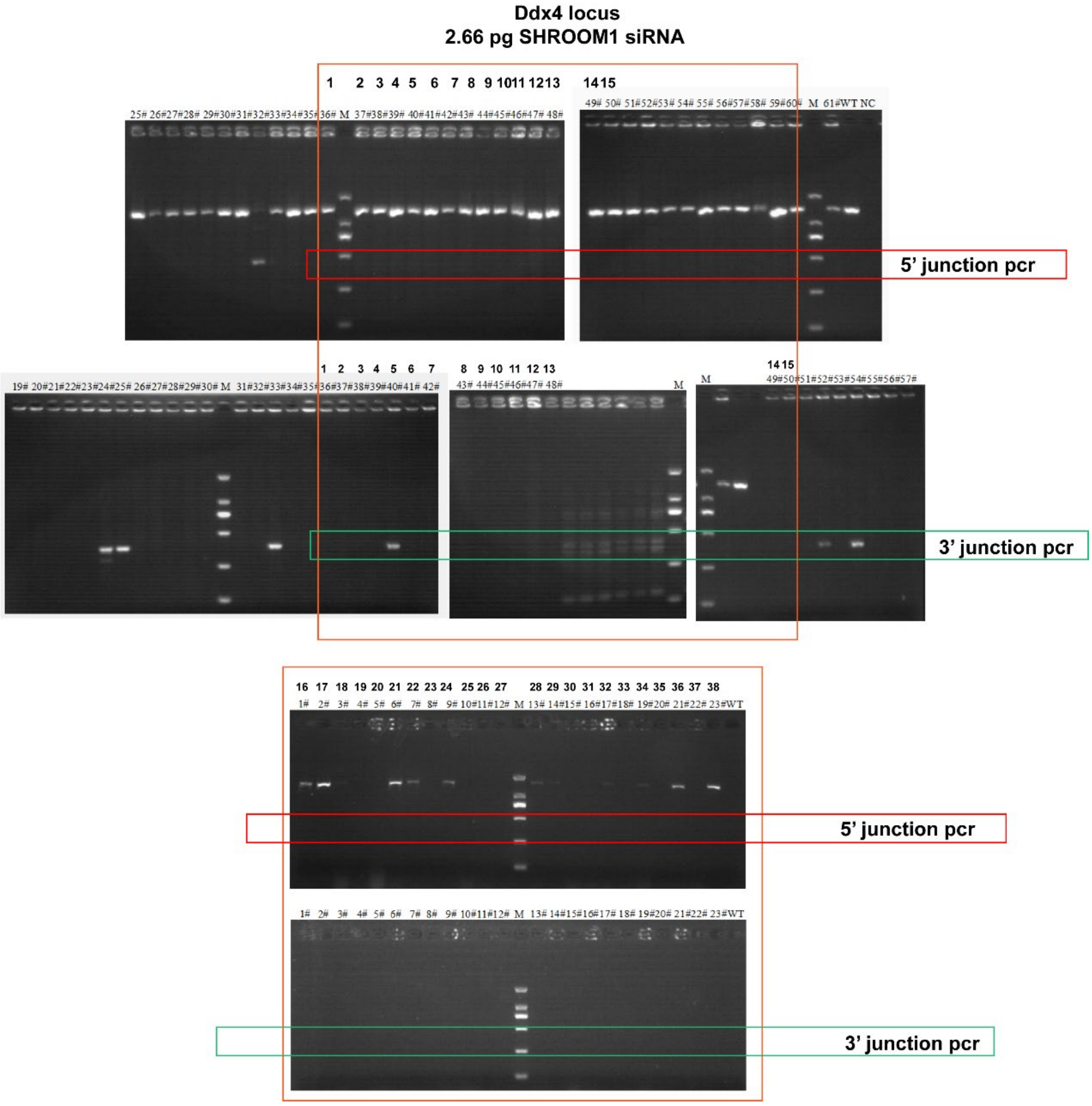

Supplementary Figure S5b

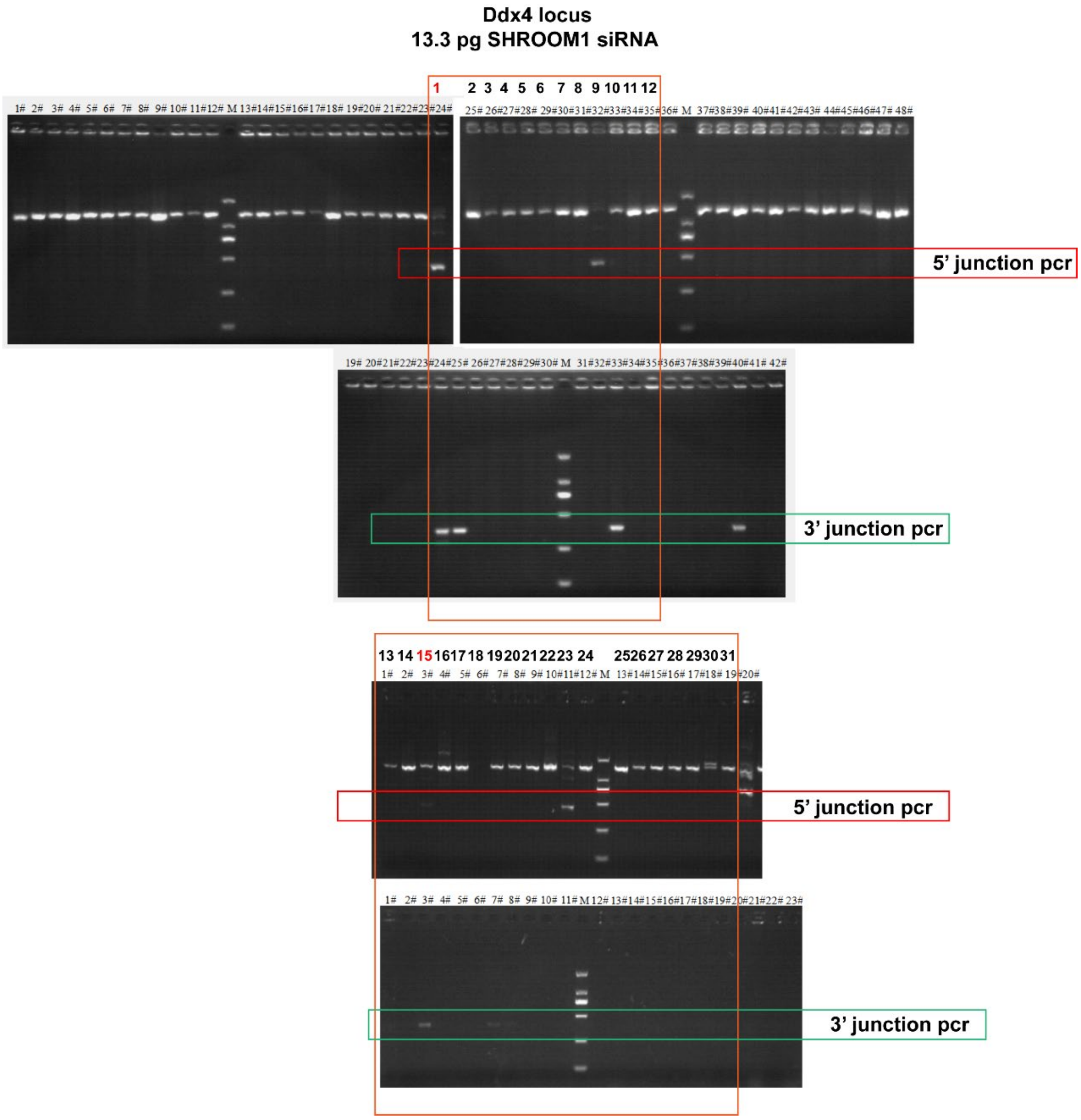

Supplementary Figure S5c

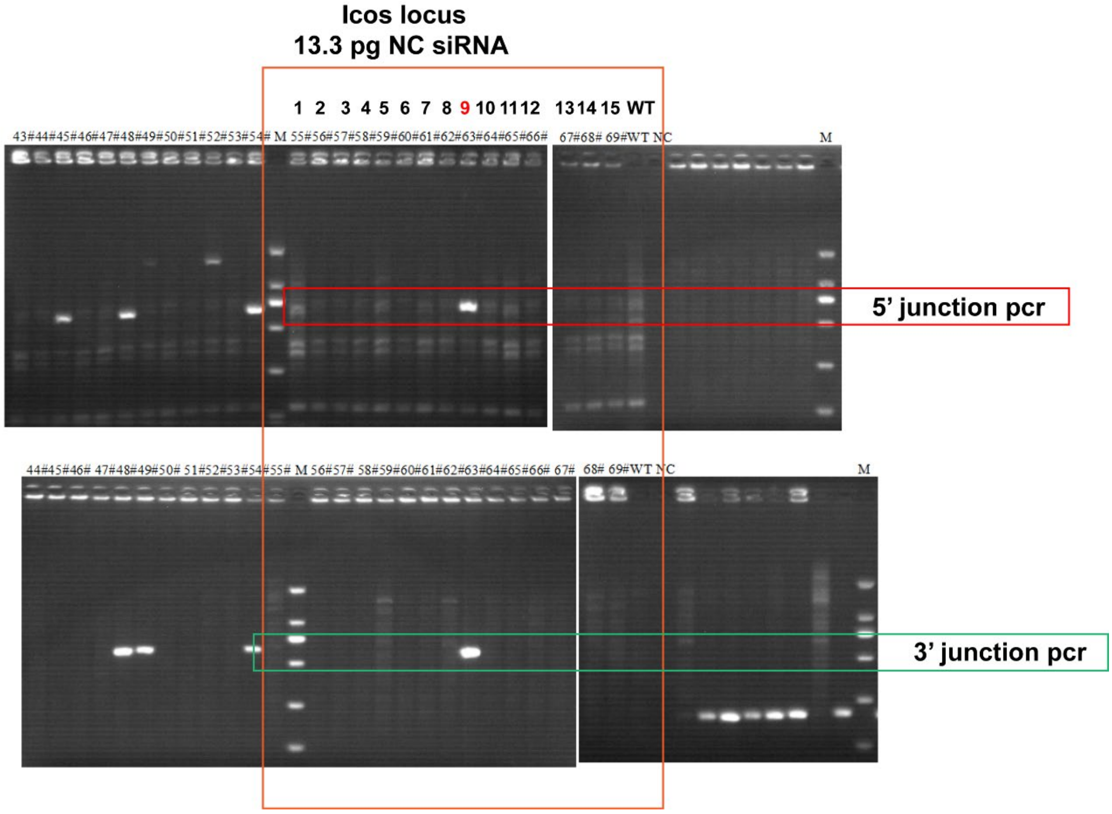

Supplementary Figure S5c

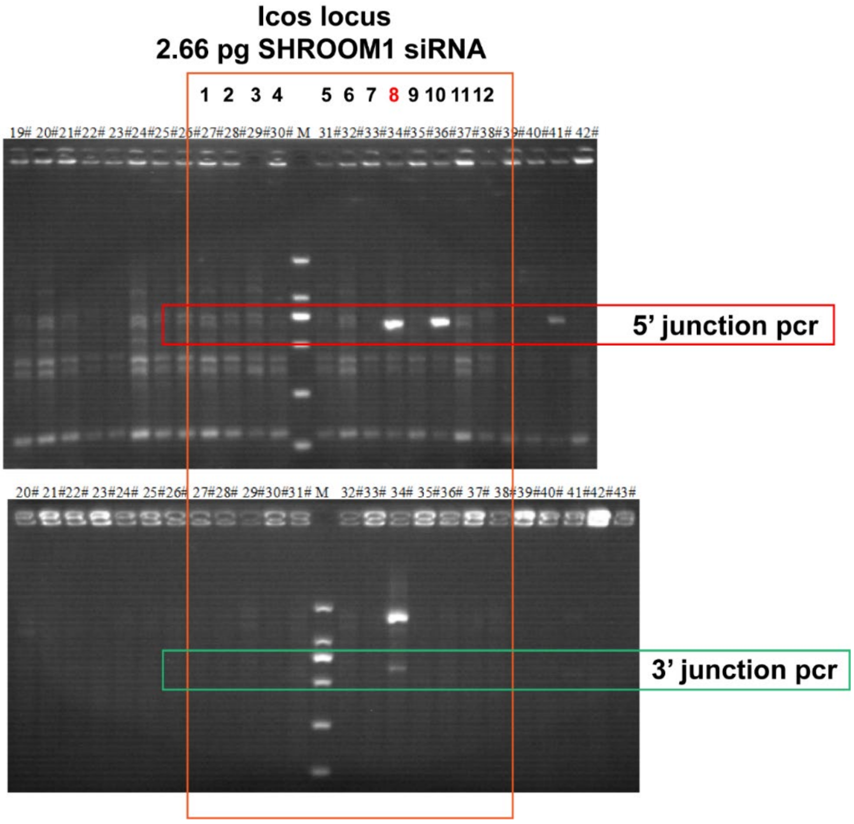

Supplementary Figure S5c

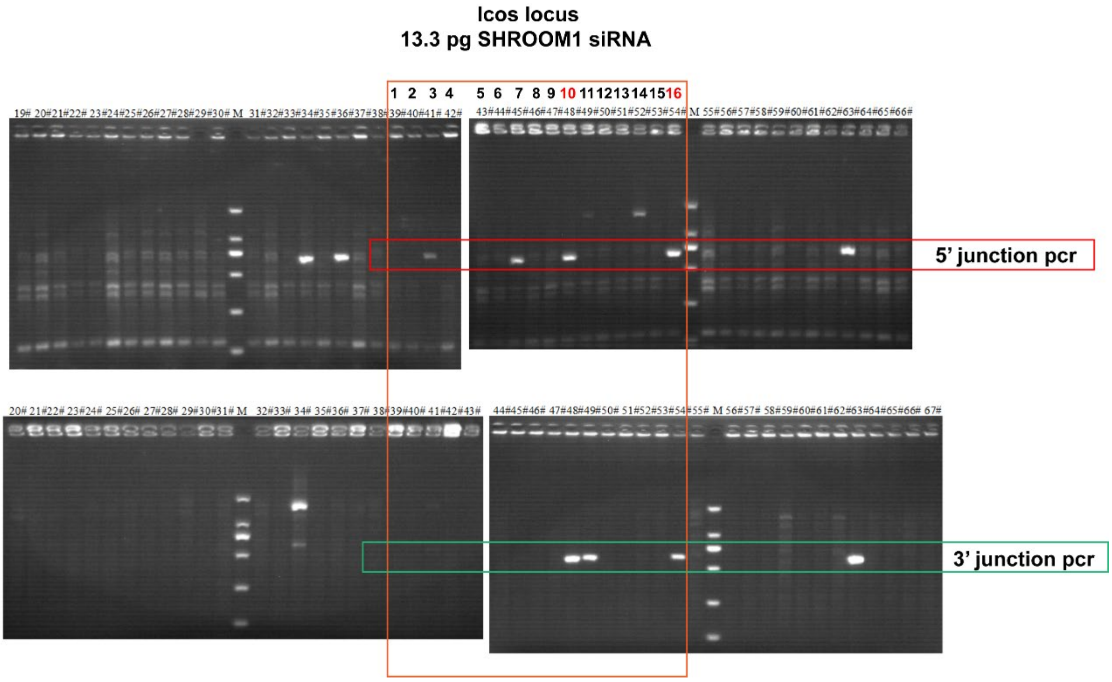

Supplement: Supplementary file 1 [file ijms-21-05821-s001.zip › ijms-862174-supplementary/Original blot figures.pdf]
