# Supplementary material for: Suppression of SHROOM1 Improves In Vitro and In Vivo Gene Integration by Promoting Homology-Directed Repair
Source: Int J Mol Sci. 2020 Aug 13;21(16):5821. doi: 10.3390/ijms21165821 (PMC7461567; doi:10.3390/ijms21165821)
Supplement: Supplementary file 1 [file ijms-21-05821-s001.zip › ijms-862174-supplementary/Supplementray data-IJMS-revision.docx]

**Supplementary Information**

Suppression of *SHROOM1* Improves In Vitro and In Vivo Gene Integration by Promoting Homology Directed Repair


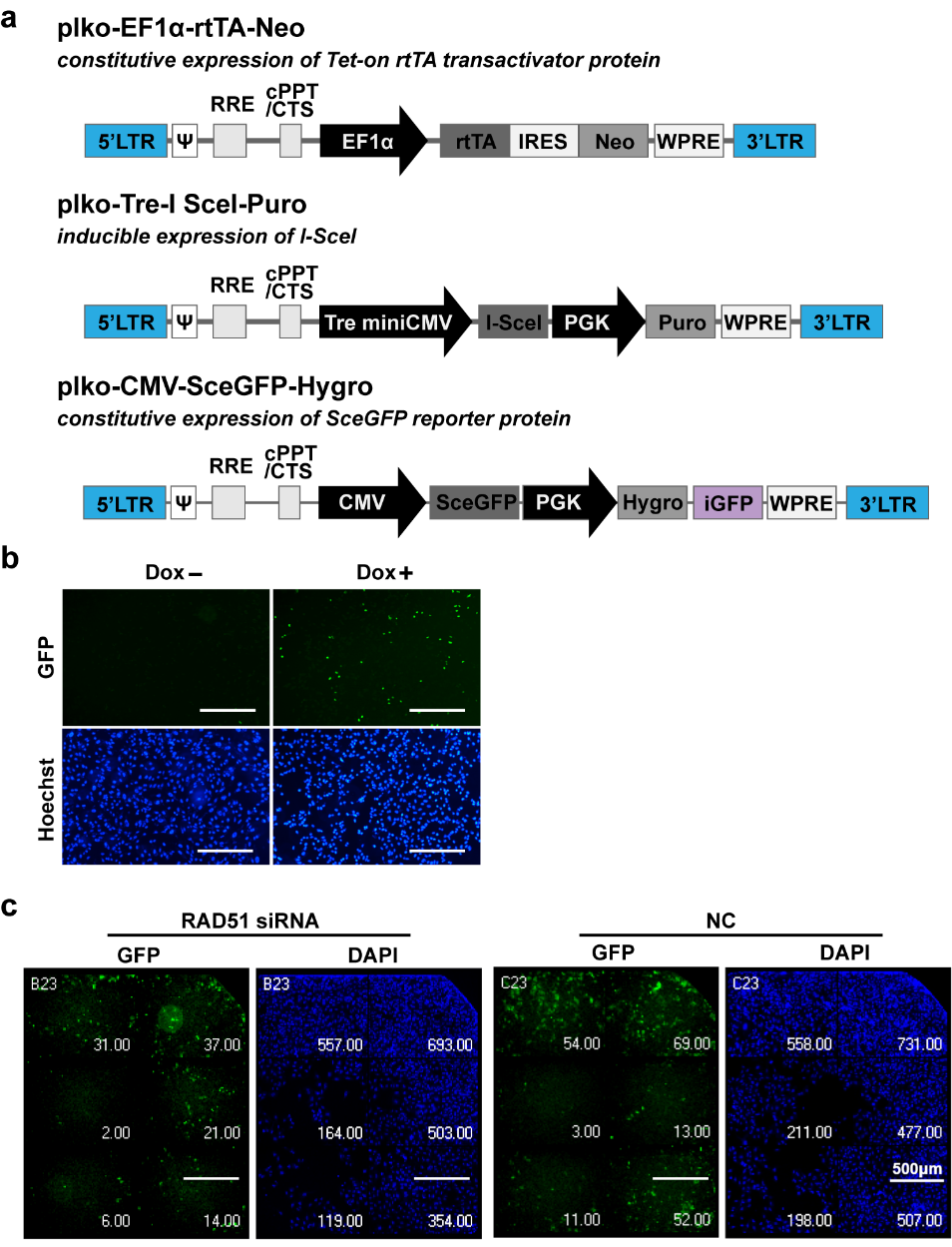
**Supplementary Figure S1.** Construction of iDR-U2OS reporter cells. (a) Schematic diagrams of lentiviral vectors used in the iDR-U2OS reporter cells; Scale bar, 200 μm. (b) HDR-mediated GFP^+^ cells generated after incision induced by *I-SceI*. Dox, doxycycline. (c) Frequencies of GFP-positive cells and total cells in each field. DAPI, 4',6-diamidino-2-phenylindole. Scale bar, 500 μm.


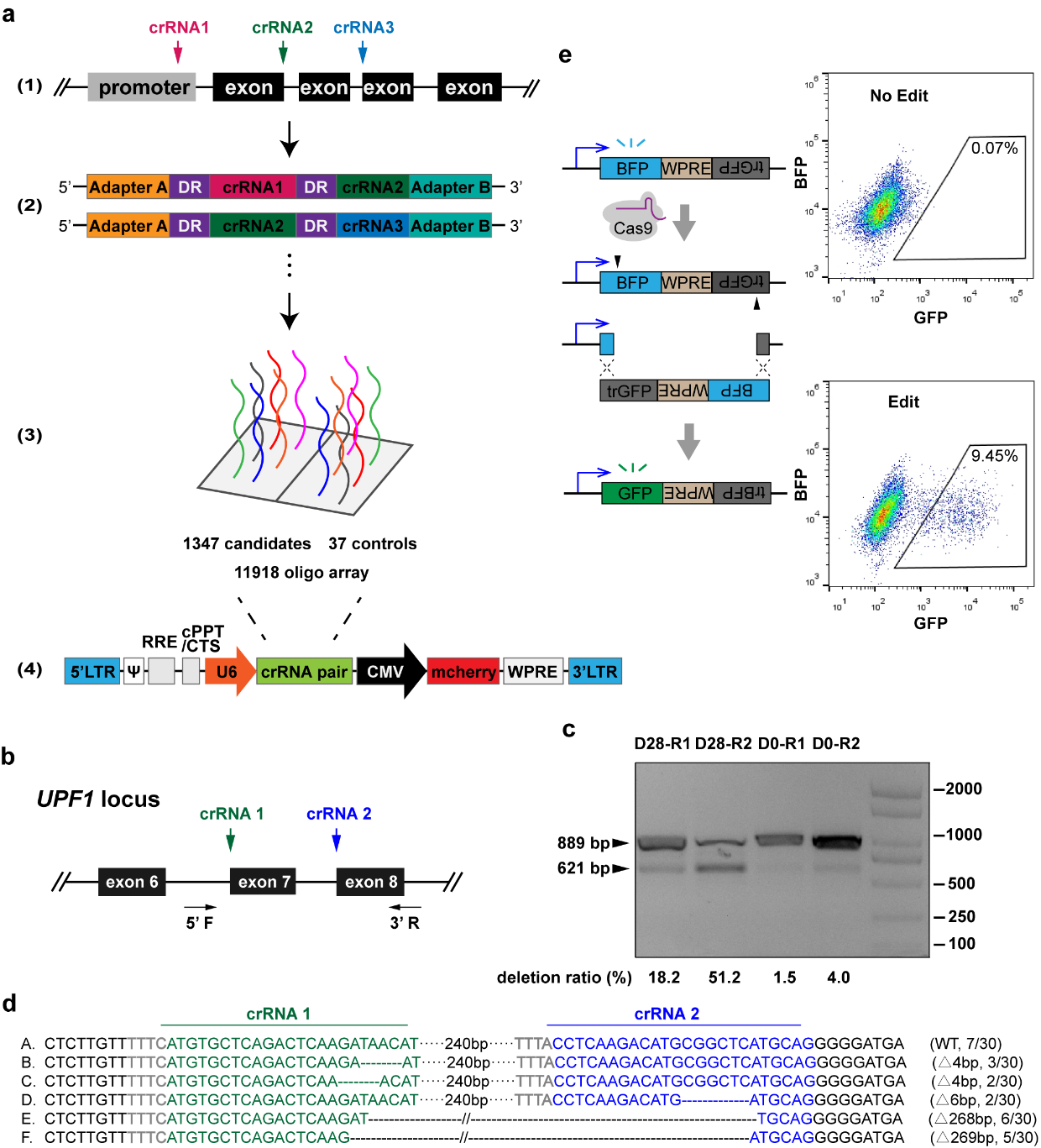


**Supplementary Figure S2.** Performances of paired crRNAs and the dual-cut BFP reporter guarantees feasibility of the screening. (a) Schematic diagrams of the paired crRNAs and library construction; two crRNAs targeting promoter or splicing sites (1) were paired randomly and combined with DR sequence to form an oligo (2). The pooled oligo array (3) consisted of 11918 oligos from 1347 candidates and 37 controls was used to amplified PCR and then integrated into a pLKO vector to form the plasmid library (4). DR, direct repeat; (b) design of crRNAs and detected primers at *UPF1* locus; F/R, primer F/R; (c) genotyping by detected primers in (b) and editing efficiencies of two independent HEK293T clones expressing one copy of paired *UPF1* crRNAs and integrated Cpf1 at day 0 and day 28; A edited band (about 621 bp) was generated from wildtype band (889 bp) after incision induced by Cpf1 and crRNAs. D0, the day of cell sorting after infection of the paired crRNA lentivirus; D28, twenty-eight days after cell sorting and lentiviral infection; R1or R2, independent experimental repeat 1 or 2; grey value was calculated by ImageJ software. (d) Sequencing results of the cocktail of shorter (621 bp) and longer (889 bp) bands of D28-R1 and D28-R2 in Figure S2c;; Triangles, deletion of nucleotides; crRNA 1, highlighted in green, crRNA 2, highlighted in blue; PAM, highlighted in grey; hollow triangle, deletion of nucleotides; (e) representative flow cytometry plots of Cpf1 expressed HEK293T cells carrying the dual-cut BFP reporter. Triangles, sgRNA target sites.


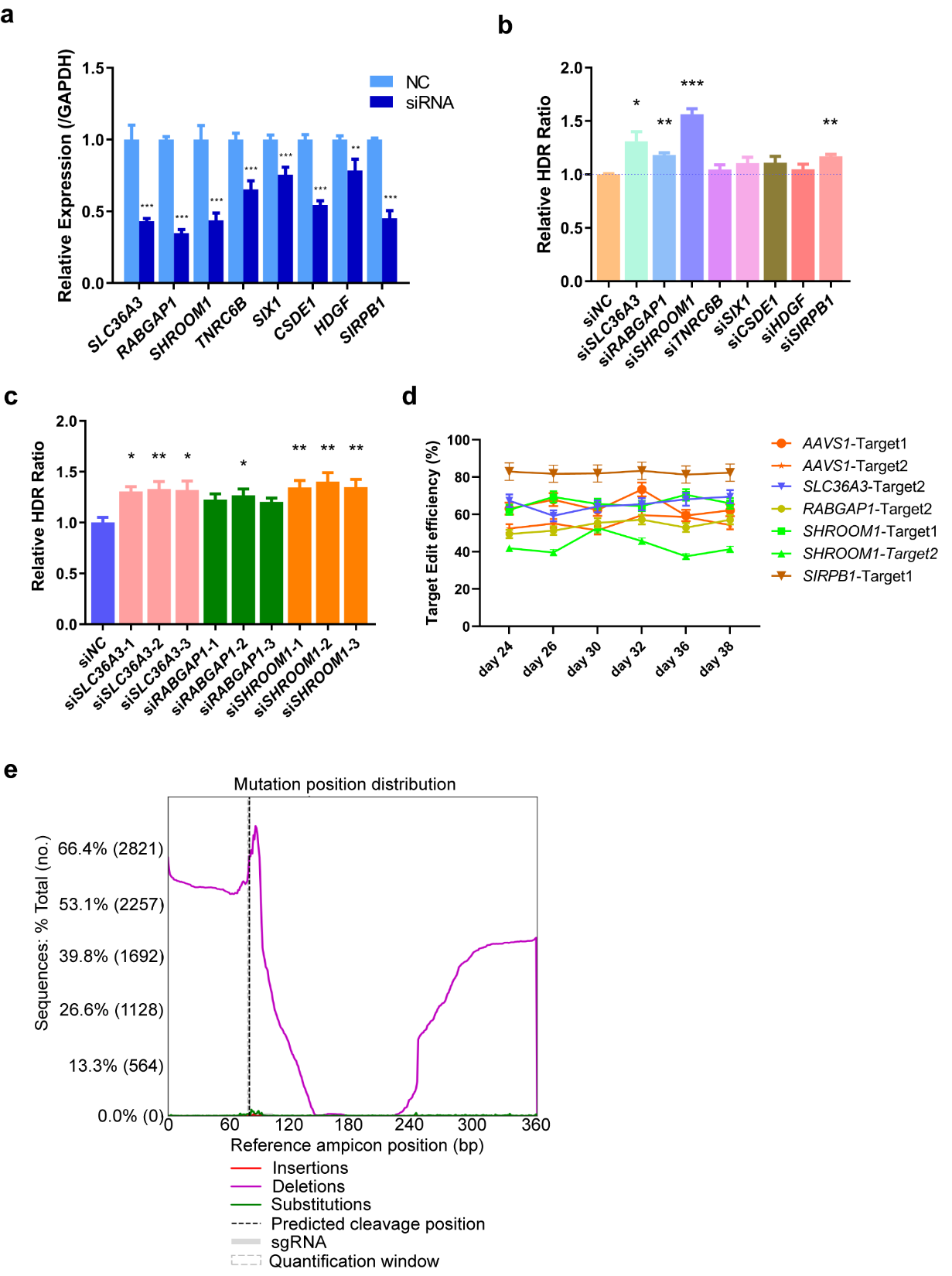


**Supplementary Figure S3.** Validation of the representative genes demonstrates the reliability of crRNA screening in dual-cut BFP reporter cells. (a) qPCR results of HEK293T cells treated individual siRNA of top eight genes enriched in the screening; Data were generated from n = 3 independent experiments. Error bars, ± SD ** P < 0.01; *** P < 0.001 by two-sided Student’s t test. (b) Relative HDR ratio of iDR-GFP U2OS reporter cells treated with individual siRNA of top eight genes enriched in the screening was detected by flow cytometry. Data were generated from n = 3 independent experiments. Error bars, ± SD * p < 0.05; ** p < 0.01; *** p < 0.001 by two-sided Student’s t-test. (c) Relative HDR ratio of the dual-cut BFP reporter cells treated with three additional siRNAs of *SLC36A3*, *RABGAP1* and *SHROOM1* was detected by flow cytometry. Data were generated from n = 3 independent experiments. Error bars, ± SD * P < 0.05; ** P < 0.01 by two-sided Student’s t test. (d) Target editing efficiency-time curve of dual-cut BFP reporter cell lines expressing mono paired crRNAs. Data were collected every two days from day 24 to day 38 after cell sorting. Data were generated from n = 3 independent experiments. (e) Mutation distribution at *SHROOM1* loci in dual-cut BFP reporter cell lines after editing for thirty days by a copy of *SHROOM1* paired crRNAs and Cpf1.


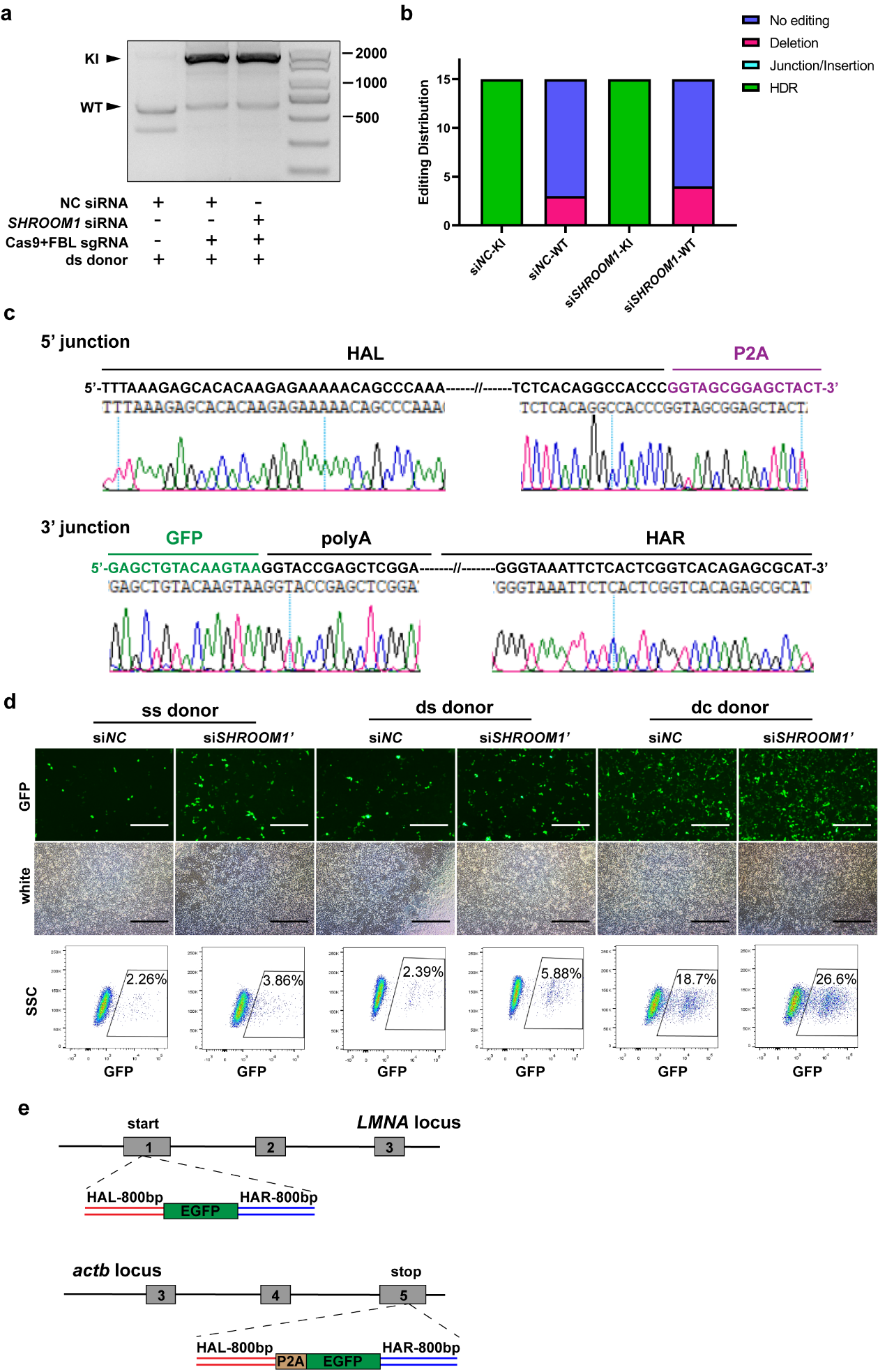


**Supplementary Figure S4.** Knockdown of *SHROOM1* mediates precise target editing after incision by CRISPR/Cas9 in vitro. (a) Genotyping of *FBL* locus in GFP+ HEK293T cells, generated by the knock-in assays and treated with NC or *SHROOM1* siRNA. WT, wildtype; KI, knock-in; NC, negative control; ds, double-strand; (b) editing distribution of the wildtype and knock-in bands of *FBL* locus in GFP+ HEK293T cells, generated by the knock-in assays, and treated with NC siRNA or *SHROOM1* siRNA; (c) sequencing results of the KI band of FBL locus in GFP+ HEK293T cells, generated by the knock-in assays, and treated with *SHROOM1* siRNA; (d) Representative visual fields and sorting charts of ss, ds, and dc donor-based strategies with *SHROOM1’* siRNA or not at the FBL locus in HEK293T cells. Scale bar, 200 μm. (e) schematic overview of the knock-in sites at human *LMNA* and moue *Actb* loci. HAL/HAR, left/right homology arm;


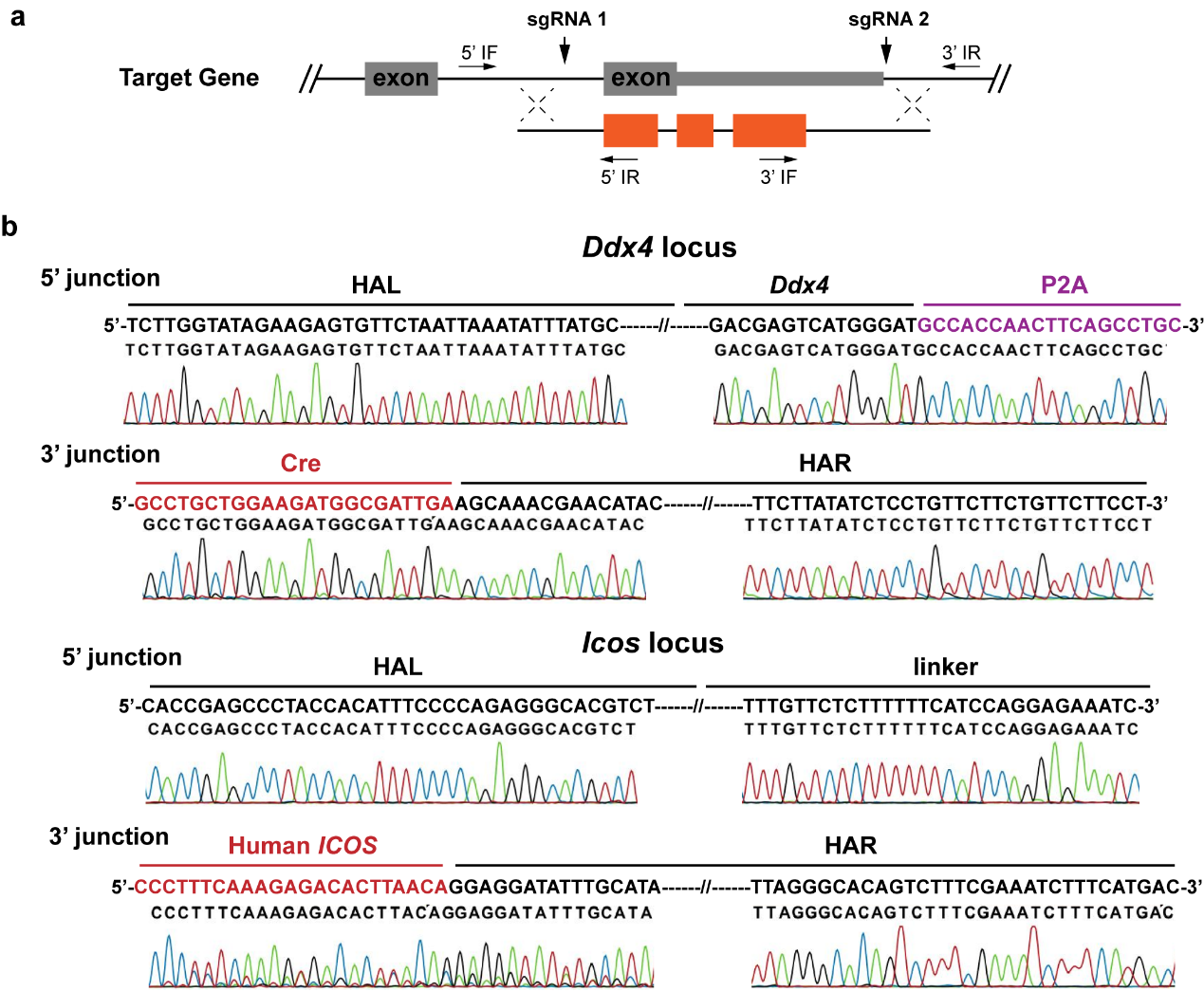


**Supplementary Figure S5.** Knockdown of *SHROOM1* promotes precise target editing after incision by CRISPR/Cas9 in mouse embryos. (a) Strategy of target integration in mouse embryos; donor with P2A linked Cre and homology arms was used at *Ddx4* locus. Donor containing human *ICOS* and homology arms was used at *Icos* locus. IF/IR, insertion forward/reverse primer; (b) sequencing results of *Ddx4* and *Icos* loci in the knock-in mice treated with *SHROOM1* siRNA after incision by CRISPR/Cas9.


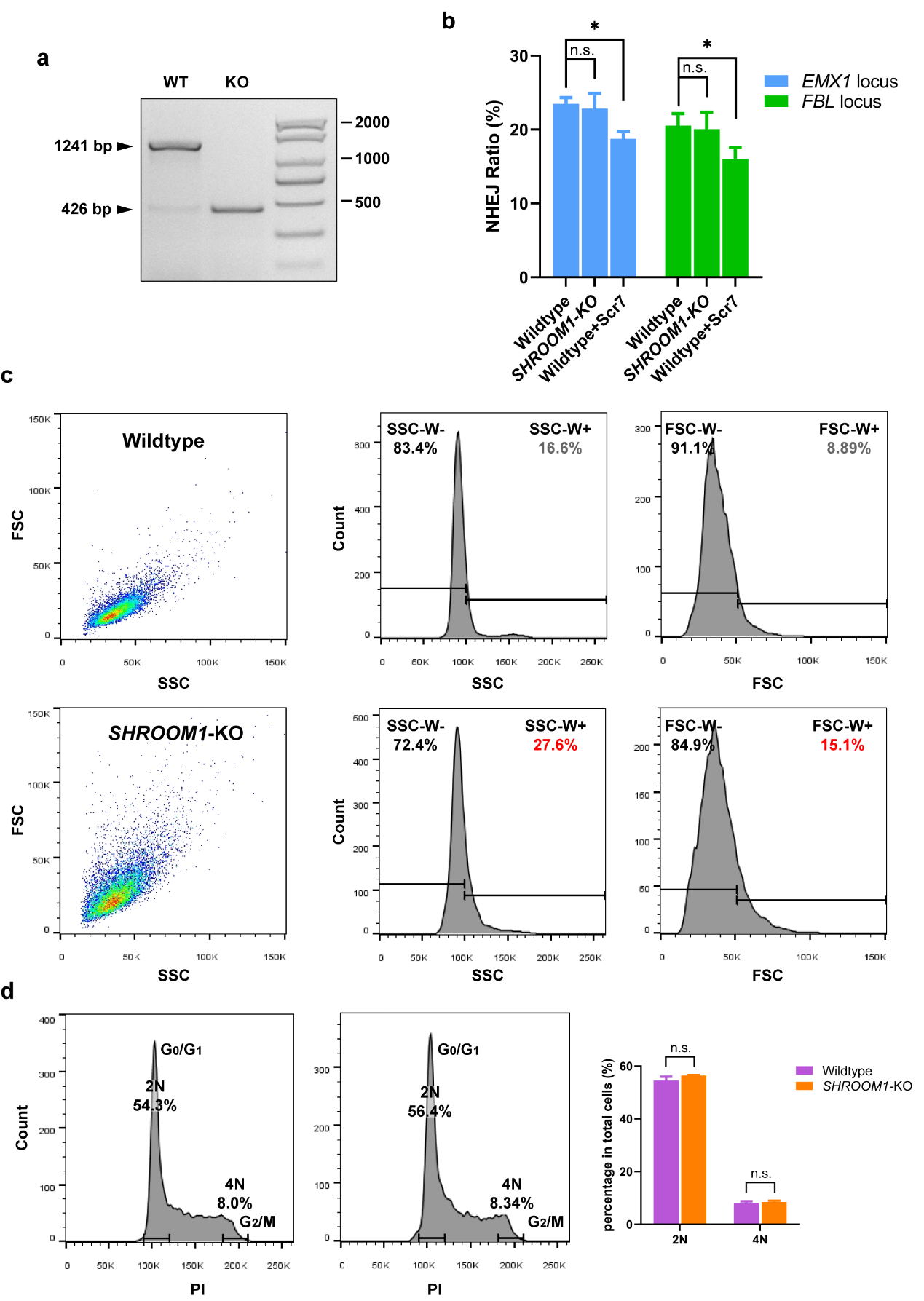


**Supplementary Figure S6.** Knockdown of *SHROOM1* has no influence on NHEJ frequency and cell cycle. (a) genotyping of *SHROOM1* in wildtype HEK293T cells and knockout HEK293T cell line. KO, knockout; WT, wildtype. (b) Deletion of *SHROOM1* did not affect NHEJ frequency at *EMX1* and *FBL* loci in HEK293T cells. (c) *SHROOM1* knockout cells shows increased FSC and SSC values comparing to wildtype HEK293T cells. In *SHROOM1* KO cells, cells with SSC > 100K make up 27.6% of total, while 16.6% in wildtype. If FSC > 50K, the proportion of *SHROOM1* knockout cells is 15.1% and higher than wildtype cells. (d) Deletion of SHROOM1 has no effect on cell cycle. Cells are stained with PI and then detect the florescence by flow cytometer. Cells at G0/G1 phase has two copy of DNA (2N). When cell stay at G2/M phase, the DNA amount comes to four copy (4N).

**Supplementary Table S1.** Gene sequences used in this study (5’-3’)

Details of the primary genes and donor sequences are as follows.

rtTA

ATGCCAAAGAGACCCAGACCCTCTAGATTAGATAAAAGTAAAGTGATTAACAGCGCATTAGAGCTGCTTAATGAGGTCGGAATCGAAGGTTTAACAACCCGTAAACTCGCCCAGAAGCTTGGTGTAGAGCAGCCTACACTGTATTGGCATGTAAAAAATAAGCGGGCTTTGCTCGACGCCTTAGCCATTGAGATGTTAGATAGGCACCATACTCACTTTTGCCCTTTAAAAGGGGAAAGCTGGCAAGATTTTTTACGCAATAACGCTAAAAGTTTTAGATGTGCTTTACTAAGTCATCGCAATGGAGCAAAAGTACATTCAGATACACGGCCTACAGAAAAACAGTATGAAACTCTCGAAAATCAATTAGCCTTTTTATGCCAACAAGGTTTTTCACTAGAGAACGCGTTATATGCACTCAGCGCTGTGGGGCATTTTACTTTAGGTTGCGTATTGGAAGATCAAGAGCATCAAGTCGCTAAAGAAGAAAGGGAAACACCTACTACTGATAGTATGCCGCCATTATTACGACAAGCTATCGAATTATTTGATCACCAAGGTGCAGAGCCAGCCTTCTTATTCGGCCTTGAATTGATCATATGCGGATTAGAAAAACAACTTAAATGTGAAAGTGGGTCCGCGTACAGCCGCGCGCGTACGAAAAACAATTACGGGTCTACCATCGAGGGCCTGCTCGATCTCCCGGACGACGACGCCCCCGAAGAGGCGGGGCTGGCGGCTCCGCGCCTGTCCTTTCTCCCCGCGGGACACACGCGCAGACTGTCGACGGCCCCCCCGACCGATGTCAGCCTGGGGGACGAGCTCCACTTAGACGGCGAGGACGTGGCGATGGCGCATGCCGACGCGCTAGACGATTTCGATCTGGACATGTTGGGGGACGGGGATTCCCCGGGTCCGGGATTTACCCCCCACGACTCCGCCCCCTACGGCGCTCTGGATATGGCCGACTTCGAGTTTGAGCAGATGTTTACCGATGCCCTTGGAATTGACGAGTACGGTGGGTAA

TRE miniCMV

Ggccaagcttcccgcgaattcctcgagtttaccactccctatcagtgatagagaaaagtgaaagtcgagtttaccactccctatcagtgatagagaaaagtgaaagtcgagtttaccactccctatcagtgatagagaaaagtgaaagtcgagtttaccactccctatcagtgatagagaaaagtgaaagtcgagtttaccactccctatcagtgatagagaaaagtgaaagtcgagtttaccactccctatcagtgatagagaaaagtgaaagtcgagtttaccactccctatcagtgatagagaaaagtgaaagtcgagctcggtacctaggcgtgtacggtgggaggcctatataagcagagctcgtttagtgaaccgtcagatcgcctctaga

SceGFP (underline: I-SceI site)

ATGGTGAGCAAGGGCGAGGAGCTGTTCACCGGGGTGGTGCCCATCCTGGTCGAGCTGGACGGCGACGTAAACGGCCACAAGTTCAGCGTGTCCGGCTAGGGATAACAGGGTAATACCTACGGCAAGCTGACCCTGAAGTTCATCTGCACCACCGGCAAGCTGCCCGTGCCCTGGCCCACCCTCGTGACCACCCTGACCTACGGCGTGCAGTGCTTCAGCCGCTACCCCGACCACATGAAGCAGCACGACTTCTTCAAGTCCGCCATGCCCGAAGGCTACGTCCAGGAGCGCACCATCTTCTTCAAGGACGACGGCAACTACAAGACCCGCGCCGAGGTGAAGTTCGAGGGCGACACCCTGGTGAACCGCATCGAGCTGAAGGGCATCGACTTCAAGGAGGACGGCAACATCCTGGGGCACAAGCTGGAGTACAACTACAACAGCCACAACGTCTATATCATGGCCGACAAGCAGAAGAACGGCATCAAGGTGAACTTCAAGATCCGCCACAACATCGAGGACGGCAGCGTGCAGCTCGCCGACCACTACCAGCAGAACACCCCCATCGGCGACGGCCCCGTGCTGCTGCCCGACAACCACTACCTGAGCACCCAGTCCGCCCTGAGCAAAGACCCCAACGAGAAGCGCGATCACATGGTCCTGCTGGAGTTCGTGACCGCCGCCGGGATCACTCTCGGCATGGACGAGCTGTACAAGTAA

iGFP (underline: arm)

AGCAAGGGCGAGGAGCTGTTCACCGGGGTGGTGCCCATCCTGGTCGAGCTGGACGGCGACGTAAACGGCCACAAGTTCAGCGTGTCCGGCGAGGGCGAGGGCGATGCCACCTACGGCAAGCTGACCCTGAAGTTCATCTGCACCACCGGCAAGCTGCCCGTGCCCTGGCCCACCCTCGTGACCACCCTGACCTACGGC

Dual-cut BFP reporter (arm: underline; sgRNA target: bold; BFP: blue; WPRE: black; BGH polyA: grey; Truncated GFP: green)

ATGGTGAGCAAGGGCGAGGAGCTGTTCACCGGGGTGGTGCCCATCCTGGTCGAGCTGGACGGCGACGTAAACGGCCACAAGTTCAGCGTGTCCGGCGAGGGCGAGGGCGATGCCACCTACGGCAAGCTGACCCTGAAGTTCATCTGCACCACCGGCAAGCTGCCCGTGCCCTGGCCCACCCTCGTGACCACCCTGA**CCCATGGCGTGCAGTGCTTCAGC**CGCTACCCCGACCACATGAAGCAGCACGACTTCTTCAAGTCCGCCATGCCCGAAGGCTACGTCCAGGAGCGCACCATCTTCTTCAAGGACGACGGCAACTACAAGACCCGCGCCGAGGTGAAGTTCGAGGGCGACACCCTGGTGAACCGCATCGAGCTGAAGGGCATCGACTTCAAGGAGGACGGCAACATCCTGGGGCACAAGCTGGAGTACAACTACAACAGCCACAACGTCTATATCATGGCCGACAAGCAGAAGAACGGCATCAAGGTGAACTTCAAGATCCGCCACAACATCGAGGACGGCAGCGTGCAGCTCGCCGACCACTACCAGCAGAACACCCCCATCGGCGACGGCCCCGTGCTGCTGCCCGACAACCACTACCTGAGCACCCAGTCCGCCCTGAGCAAAGACCCCAACGAGAAGCGCGATCACATGGTCCTGCTGGAGTTCGTGACCGCCGCCGGGATCACTCTCGGCATGGACGAGCTGTACAAGtaagaattcgtcgagggacctaataacttcgtatagcatacattatacgaagttatacatgtttaagggttccggttccactaggtacaattcgatatcaagcttatcgataatcaacctctggattacaaaatttgtgaaagattgactggtattcttaactatgttgctccttttacgctatgtggatacgctgctttaatgcctttgtatcatgctattgcttcccgtatggctttcattttctcctccttgtataaatcctggttgctgtctctttatgaggagttgtggcccgttgtcaggcaacgtggcgtggtgtgcactgtgtttgctgacgcaacccccactggttggggcattgccaccacctgtcagctcctttccgggactttcgctttccccctccctattgccacggcggaactcatcgccgcctgccttgcccgctgctggacaggggctcggctgttgggcactgacaattccgtggtgttgtcggggaaatcatcgtcctttccttggctgctcgcctgtgttgccacctggattctgcgcgggacgtccttctgctacgtcccttcggccctcaatccagcggaccttccttcccgcggcctgctgccggctctgcggcctcttccgcgtcttcgccttcgccctcagacgagtcggatctccctttgggccgcctccccgcatcgataccgtcgacctcgatcgagacctagaaaaacatggagcaatcacaagtaggaattcCTGTGCCTTCTAGTTGCCAGCCATCTGTTGTTTGCCCCTCCCCCGTGCCTTCCTTGACCCTGGAAGGTGCCACTCCCACTGTCCTTTCCTAATAAAATGAGGAAATTGCATCGCATTGTCTGAGTAGGTGTCATTCTATTCTGGGGGGTGGGGTGGGGCAGGACAGCAAGGGGGAGGATTGGGAAGACAATAGCAGGCATGCTGGGGATGCGGTGGGCTCTATGGTATCATTCTTACTTGTACAGCTCGTCCATGCCGAGAGTGATCCCGGCGGCGGTCACGAACTCCAGCAGGACCATGTGATCGCGCTTCTCGTTGGGGTCTTTGCTCAGGGCGGACTGGGTGCTCAGGTAGTGGTTGTCGGGCAGCAGCACGGGGCCGTCGCCGATGGGGGTGTTCTGCTGGTAGTGGTCGGCGAGCTGCACGCTGCCGTCCTCGATGTTGTGGCGGATCTTGAAGTTCACCTTGATGCCGTTCTTCTGCTTGTCGGCCATGATATAGACGTTGTGGCTGTTGTAGTTGTACTCCAGCTTGTGCCCCAGGATGTTGCCGTCCTCCTTGAAGTCGATGCCCTTCAGCTCGATGCGGTTCACCAGGGTGTCGCCCTCGAACTTCACCTCGGCGCGGGTCTTGTAGTTGCCGTCGTCCTTGAAGAAGATGGTGCGCTCCTGGACGTAGCCTTCGGGCATGGCGGACTTGAAGAAGTCGTGCTGCTTCATGTGGTCGGGGTAGCGGCTGAAGCACTGCACGCCGTAGGTCAGGGTGGTCACGAGGGTGGGCCAGGGCACGGGCAGCTTGCCGGTGGTGCAGATGAACTTCAGGGTCAGCTTGCCGTAGGTGGCATCGCCCTCGCCCTCGCCGGACACGCTGAACTTGTGGCCGTTTACGTCGCCGTCCAGCTCGACCAGGATGGGCACCACCCCGGTGAACAGCTCCTCGCCCTTGCTCACTCA**GCTGAAGCACTGCACGCCATGGG**GTCCTCCTTGAAGTCGATGCCCTTCAGCTCGATGCGGTTCACCAGGGTGTCGCCCTCGAACTTCACCTCGGCGCGGGTCTTGTAGTTGCCGTCGTCCTTGAAGAAGATGGTGCGCTCCTGGACGTAGCCTTCGGGCATGGCGGACTTGAAGAAGTCGTGCTGCTTCATGTGGTCGGGGTAGCGGCTGAAGCACTGCACGC

*FBL* double cut donor (arm: underline; sgRNA target: bold; P2A: red; GFP: green; BGH polyA: grey)

**CTCTCACAGGCCACCCCCCAAGG**GTCTCAATCTCCTGATTTCGTGATTGAGCCACCTCGGCCTCCCAAAGTGCTGGGATTACAGGCGTGAGCCACCACGCCCAGCCTTAGACTGGGTAATTTATAATGAATGGAAATTTATTTGGCTCCCAGTTCCAAAGGCTGGAAAGTCCAAGATTGGAGGTCTGAATCTGGCGAGGGCCTTCTTGCTGTCATCCATTGGCAGAAGGGTGAGAGCAAGATAGAAAGGGGGCATAATCATCCTTTTAATCAGCAACCCACTCTTGTGATAATAGCATTACTCTATTCAGGAAGGCAGAGGCCTCATGACCTGAATCATCTCTCGAAGGTCCCACCTCTCAACTCTTGCATTTAAGGGTTACGTTTCCAACACATGAACTTTGGGGGACACACTAGAACCATAGCACTGAGTTTTACTTGAATTAATAATGAAAACATCTGGTTTAAAGAGCACACAAGAGAAAAACAGCCCAAAGCCCTGTTGTAGACATTAGTCCTTTCTCCTCTTTAGGCCAACTGCATTGACTCCACAGCCTCAGCCGAGGCCGTGTTTGCCTCCGAAGTGAAAAAGATGCAACAGGAGAACATGAAGCCGCAGGAGCAGTTGACCCTTGAGCCATATGAAAGAGACCATGCCGTGGTCGTGGGAGTGTACAGGTGAGCAGGGGCCCAGCAATACACCAAGACAGACATCTCTGTCCCTTGCACCCCGAGTGCCATGATCCTGGGGACCCTCCTTCATCACCTATCTTCCTCTCACAGGCCACCCGGTAGCGGAGCTACTAACTTCAGCCTGCTGAAGCAGGCTGGAGACGTGGAGGAGAACCCTGGACCTGCCACCATGGTGAGCAAGGGCGAGGAGCTGTTCACCGGGGTGGTGCCCATCCTGGTCGAGCTGGACGGCGACGTAAACGGCCACAAGTTCAGCGTGTCCGGCGAGGGCGAGGGCGATGCCACCTACGGCAAGCTGACCCTGAAGTTCATCTGCACCACCGGCAAGCTGCCCGTGCCCTGGCCCACCCTCGTGACCACCCTGACCTACGGCGTGCAGTGCTTCAGCCGCTACCCCGACCACATGAAGCAGCACGACTTCTTCAAGTCCGCCATGCCCGAAGGCTACGTCCAGGAGCGCACCATCTTCTTCAAGGACGACGGCAACTACAAGACCCGCGCCGAGGTGAAGTTCGAGGGCGACACCCTGGTGAACCGCATCGAGCTGAAGGGCATCGACTTCAAGGAGGACGGCAACATCCTGGGGCACAAGCTGGAGTACAACTACAACAGCCACAACGTCTATATCATGGCCGACAAGCAGAAGAACGGCATCAAGGTGAACTTCAAGATCCGCCACAACATCGAGGACGGCAGCGTGCAGCTCGCCGACCACTACCAGCAGAACACCCCCATCGGCGACGGCCCCGTGCTGCTGCCCGACAACCACTACCTGAGCACCCAGTCCGCCCTGAGCAAAGACCCCAACGAGAAGCGCGATCACATGGTCCTGCTGGAGTTCGTGACCGCCGCCGGGATCACTCTCGGCATGGACGAGCTGTACAAGTAAGGTACCGAGCTCGGATCCACTAGTCCAGTGTGGTGGAATTCTGCAGATATCCAGCACAGTGGCGGCCGCTCGACTAGAGGGCCCGTTTAAACCCGCTGATCAGCCTCGACTGTGCCTTCTAGTTGCCAGCCATCTGTTGTTTGCCCCTCCCCCGTGCCTTCCTTGACCCTGGAAGGTGCCACTCCCACTGTCCTTTCCTAATAAAATGAGGAAATTGCATCGCATTGTCTGAGTAGGTGTCATTCTATTCTGGGGGGTGGGGTGGGGCAGGACAGCAAGGGGGAGGATTGGGAAGACAATAGCAGGCATGCTGGGGATGCGGTGGGCTCTATGGCTTCTGAGGCGGAAAGAACCAGCCCAAGGTGAAGAACTGAAGTTCAGCGCTGTCAGGATTGCGAGAGATGTGTGTTGATACTGTTGCACGTGTGTTTTTCTATTAAAAGACTCATCCGTCTCCCATGTCTGCTGCTCATTCCTCCCCTTGACCTGCTGACACAGGGAGCACGCACCCTTGGTCAATTTTGCGGGGTTGGGTAAATTCTCACTCGGTCACAGAGCGCATGCTCCGTTTCTAGCTGCCTTTGCGCAGCGGCAGCCTGGATTTCGGTTCTTGGGTGGGATTGGTAGCTCGCTGCGCATGCGTGCAGGTAAGCGGCCATCTCGCGCAGGCGGAGTGTCAGTGTGGGTCACGTGAGGGGAGCGGAGAGGGAGGGATGGGGGCGGAGTCCAGGGCGTGGGGGGGCCGGTTTGTTGTGGTCGCCATTTTGCTGGTTGCATTACTGGGTAATCGGGGCCCTGGCTTGCCGCGTCCGCCGGATACCCTCAGCCAGTGGGCAGGTCTGAGCTCGGGCTCCCCGAGCAGTTTGAGTCCCCTTGCCCGCTCCTTCAGGTAACGGCGCGGGGACGGGTGGGGCGGCAAGCGGTCGCAGGGAGGTGGGCAGGACGGGATCCGCCCTGCTCCCGTCGCCGTGAGACTTAGCACGAGGCCAAGGGAGGAGAGGAGGGGGGTGGCAGGCAGGTGCGGGCCCTGCCTGGCTATTCATAGTTGAATTCCTGGAACCGGCCAAGCCCGAGGAAGCAGTTGCAGGAGGGAGGCTGGGAGGGGGTAGCCGGGCCCCACTCCCGCCCTTTGTTTGGGCTCAGCTCCGCGGGCCGCTTCTTCGTCGCCTAGC**CCTTGGGGGGTGGCCTGTGAGAG**

*LMNA* double cut donor (arm: underline; sgRNA target: bold; GFP: green)

**CCATGGAGACCCCGTCCCAGCGG**GGGGACTGTGGCTTGTTGCTTGGGTCTAAAAACGAATGCTTGGCTTTGAAGAGAGATAGATTGGGGCAAAAGAAAGAAAAAAAGGGACCCCCCAAACTCCTTGATCCCTGGCCCCAAACTGGGGGCATAAAGGAACTCAGGTTCCAGAACTTTGCTCCCCCCAGGGAACCCAGGCATTCCTTCTCCACCCCACTCCTGGCACACTGAGATGCAGCTCTGAATGGGCTGCCCACGTGTGGAGGGGGGTTGGGGTGACTCACTATTACTACTGGGAGGACAGGGGGAGCCAGTGGTGGAAGAAGGGTGAGTCACACTGATGGGCACCAGCCTCAGCCCTCCCCCCACTTTCCTGGCTCCCAGCCCTGCCTACCTGACCCTCTCCCTTGCTTTGCGCCCACTTCCCTCTCTTTCTCCCCGACCCTTTTGCCCACCCACTCTCCCTCCTTGGCTCTGCCCTCTAGCCCAGAAGGTCTGAGGCAATGGGGGCAAGCTTGGAGCCGACAGTGCTGAGCAGGCAGGAGCCAAGAGAGGGGAAGCTTGAGCCTCACGCAGTTAGGGGTGCGCTGGAGAGGGTGGGGCCCGACTCCGCCACACCCCAACGGTCCTTCCCCCTCCTCACCACTCCCGCCCCCACCCCCAATGGATCTGGGACTGCCCCTTTAAGAGTAGTGGCCCCTCCTCCCTTCAGAGGAGGACCTATTAGAGCCTTTGCCCCGGCGTCGGTGACTCAGTGTTCGCGGGAGCGCCGCACCTACACCAGCCAACCCAGATCCCGAGGTCCGACAGCGCCCGGCCCAGATCCCCACGCCTGCCAGGAGCAAGCCGAGAGCCAGCCGGCCGGCGCACTCCGACTCCGAGCAGTCTCTGTCCTTCGACCCGAGCCCCGCGCCCTTTCCGGGACCCCTGCCCCGCGGGCAGCGCTGCCAACCTGCCGGCCATGGAGACCCCGTCCGTGAGCAAGGGCGAGGAGCTGTTCACCGGGGTGGTGCCCATCCTGGTCGAGCTGGACGGCGACGTAAACGGCCACAAGTTCAGCGTGTCCGGCGAGGGCGAGGGCGATGCCACCTACGGCAAGCTGACCCTGAAGTTCATCTGCACCACCGGCAAGCTGCCCGTGCCCTGGCCCACCCTCGTGACCACCCTGACCTACGGCGTGCAGTGCTTCAGCCGCTACCCCGACCACATGAAGCAGCACGACTTCTTCAAGTCCGCCATGCCCGAAGGCTACGTCCAGGAGCGCACCATCTTCTTCAAGGACGACGGCAACTACAAGACCCGCGCCGAGGTGAAGTTCGAGGGCGACACCCTGGTGAACCGCATCGAGCTGAAGGGCATCGACTTCAAGGAGGACGGCAACATCCTGGGGCACAAGCTGGAGTACAACTACAACAGCCACAACGTCTATATCATGGCCGACAAGCAGAAGAACGGCATCAAGGTGAACTTCAAGATCCGCCACAACATCGAGGACGGCAGCGTGCAGCTCGCCGACCACTACCAGCAGAACACCCCCATCGGCGACGGCCCCGTGCTGCTGCCCGACAACCACTACCTGAGCACCCAGTCCGCCCTGAGCAAAGACCCCAACGAGAAGCGCGATCACATGGTCCTGCTGGAGTTCGTGACCGCCGCCGGGATCACTCTCGGCATGGACGAGCTGTACAAGCAGCGGCGCGCCACCCGCAGCGGGGCGCAGGCCAGCTCCACTCCGCTGTCGCCCACCCGCATCACCCGGCTGCAGGAGAAGGAGGACCTGCAGGAGCTCAATGATCGCTTGGCGGTCTACATCGACCGTGTGCGCTCGCTGGAAACGGAGAACGCAGGGCTGCGCCTTCGCATCACCGAGTCTGAAGAGGTGGTCAGCCGCGAGGTGTCCGGCATCAAGGCCGCCTACGAGGCCGAGCTCGGGGATGCCCGCAAGACCCTTGACTCAGTAGCCAAGGAGCGCGCCCGCCTGCAGCTGGAGCTGAGCAAAGTGCGTGAGGAGTTTAAGGAGCTGAAAGCGCGGTGAGTTCGCCCAGGTGGCTGCGTGCCTGGCGGGGAGTGGAGAGGGCGGCGGGCCGGCGCCCCTGGCCGGCCGCAGGAAGGGAGTGAGAGGGCCTGGAGGCCGATAACTTTGCCATAGTCTCCTCCCTCCCCGGAACTGCCCCCAGCGGGTGACTGGCAGTGTCAAGGGGAATTGTCAAGACAGGACAGAGAGGGAAGTGGTGGTCTCTGGGAGAGGGTCGGGGAGGATATAAGGAATGGTGGGGGTATCAGGGACAAGTTGGGGCTGGGGCCGGCCTGAATTCGGTCAGATTGGGATTTGCCAACTATTTGGAGCCGGGGGGAGGGGCTTGAGCAAAACAGAACTAGCCCTGCCAGCTCGAAGAACTCTGGGCACCCAGGACACATCGGAGTGGCAGAAAGGGTCCTGTTAGAACTTTGTTAGCGGGCTTGGCAC**CCGCTGGGACGGGGTCTCCATGG**

*Actb* double cut donor (arm: underline; sgRNA target: bold; P2A: red; GFP: green; BGH polyA: grey)

**AGTCCGCCTAGAAGCACTTGCGG**GGCTGGCCGGGACCTGACAGACTACCTCATGAAGATCCTGACCGAGCGTGGCTACAGCTTCACCACCACAGCTGAGAGGGAAATCGTGCGTGACATCAAAGAGAAGCTGTGCTATGTTGCTCTAGACTTCGAGCAGGAGATGGCCACTGCCGCATCCTCTTCCTCCCTGGAGAAGAGCTATGAGCTGCCTGACGGCCAGGTCATCACTATTGGCAACGAGCGGTTCCGATGCCCTGAGGCTCTTTTCCAGCCTTCCTTCTTGGGTAAGTTGTAGCCTAGTCCTTTCTCCATCTAAAGGTGACAAAACTCCTGAGGCCATAGTACAAGTTAAGTCTGATTTCTGTCACTCTTCTCTTAGGTATGGAATCCTGTGGCATCCATGAAACTACATTCAATTCCATCATGAAGTGTGACGTTGACATCCGTAAAGACCTCTATGCCAACACAGTGCTGTCTGGTGGTACCACCATGTACCCAGGCATTGCTGACAGGATGCAGAAGGAGATTACTGCTCTGGCTCCTAGCACCATGAAGATCAAGGTAAGCTAAGCATCCTTAGCTTGGTGAGGGTGGGCCCTGTGGTTGTCAGAGCAACCTTCTAGGTTTAAGGGGAATCCCAGCACCCAGAGAGCTCACCATTCACCATCTTGTCTTGCTTTCTTCAGATCATTGCTCCTCCTGAGCGCAAGTACTCTGTGTGGATCGGTGGCTCCATCCTGGCCTCACTGTCCACCTTCCAGCAGATGTGGATCAGCAAGCAGGAGTACGATGAGTCCGGCCCCTCCATCGTGCACCGCAAGTGCTTCGGTAGCGGAGCTACTAACTTCAGCCTGCTGAAGCAGGCTGGAGACGTGGAGGAGAACCCTGGACCTGCCACCATGGTGAGCAAGGGCGAGGAGCTGTTCACCGGGGTGGTGCCCATCCTGGTCGAGCTGGACGGCGACGTAAACGGCCACAAGTTCAGCGTGTCCGGCGAGGGCGAGGGCGATGCCACCTACGGCAAGCTGACCCTGAAGTTCATCTGCACCACCGGCAAGCTGCCCGTGCCCTGGCCCACCCTCGTGACCACCCTGACCTACGGCGTGCAGTGCTTCAGCCGCTACCCCGACCACATGAAGCAGCACGACTTCTTCAAGTCCGCCATGCCCGAAGGCTACGTCCAGGAGCGCACCATCTTCTTCAAGGACGACGGCAACTACAAGACCCGCGCCGAGGTGAAGTTCGAGGGCGACACCCTGGTGAACCGCATCGAGCTGAAGGGCATCGACTTCAAGGAGGACGGCAACATCCTGGGGCACAAGCTGGAGTACAACTACAACAGCCACAACGTCTATATCATGGCCGACAAGCAGAAGAACGGCATCAAGGTGAACTTCAAGATCCGCCACAACATCGAGGACGGCAGCGTGCAGCTCGCCGACCACTACCAGCAGAACACCCCCATCGGCGACGGCCCCGTGCTGCTGCCCGACAACCACTACCTGAGCACCCAGTCCGCCCTGAGCAAAGACCCCAACGAGAAGCGCGATCACATGGTCCTGCTGGAGTTCGTGACCGCCGCCGGGATCACTCTCGGCATGGACGAGCTGTACAAGTAAGGTACCGAGCTCGGATCCACTAGTCCAGTGTGGTGGAATTCTGCAGATATCCAGCACAGTGGCGGCCGCTCGACTAGAGGGCCCGTTTAAACCCGCTGATCAGCCTCGACTGTGCCTTCTAGTTGCCAGCCATCTGTTGTTTGCCCCTCCCCCGTGCCTTCCTTGACCCTGGAAGGTGCCACTCCCACTGTCCTTTCCTAATAAAATGAGGAAATTGCATCGCATTGTCTGAGTAGGTGTCATTCTATTCTGGGGGGTGGGGTGGGGCAGGACAGCAAGGGGGAGGATTGGGAAGACAATAGCAGGCATGCTGGGGATGCGGTGGGCTCTATGGCTTCTGAGGCGGAAAGAACCAGCTAGGCGGACTGTTACTGAGCTGCGTTTTACACCCTTTCTTTGACAAAACCTAACTTGCGCAGAAAAAAAAAAAATAAGAGACAACATTGGCATGGCTTTGTTTTTTTAAATTTTTTTTAAAGTTTTTTTTTTTTTTTTTTTTTTTTTTTTTAAGTTTTTTTGTTTTGTTTTGGCGCTTTTGACTCAGGATTTAAAAACTGGAACGGTGAAGGCGACAGCAGTTGGTTGGAGCAAACATCCCCCAAAGTTCTACAAATGTGGCTGAGGACTTTGTACATTGTTTTGTTTTTTTTTTTTTTTGGTTTTGTCTTTTTTTAATAGTCATTCCAAGTATCCATGAAATAAGTGGTTACAGGAAGTCCCTCACCCTCCCAAAAGCCACCCCCACTCCTAAGAGGAGGATGGTCGCGTCCATGCCCTGAGTCCACCCCGGGGAAGGTGACAGCATTGCTTCTGTGTAAATTATGTACTGCAAAAATTTTTTTAAATCTTCCGCCTTAATACTTCATTTTTGTTTTTAATTTCTGAATGGCCCAGGTCTGAGGCCTCCCTTTTTTTTGTCCCCCCAACTTGATGTATGAAGGCTTTGGTCTCCCTGGGAGGGGGTTGAGGTGTTGAGGCAGCCAGGGCTGGCCTGTACACTGACTTGAGACCAATAAAAGTGCACACCTTACCTTACACAAACAGCTTGTGGCTCTGTGGCTTTGCTGGGTGTGGGGAGCAGGTTGGGTGGGTGTGGAGCTCTATTGGGGGGGGCATCTAGGGTGGGCTAGGCCTTGCTGATGGTATCTAGTGGGAGG**CCGCAAGTGCTTCTAGGCGGACT**

*Ddx4* ss donor (arm: underline)

CAGTGAATCTTGGTATAGAAGAGTGTTCTAATTAAATATTTATGCCAATTTGAAGTATAATAAAATTTTCAGCTCTCTAAAAGCTTGTGACTTGAAAAAATCATTTTCTTCAGTCATTTTGAACAGTGTAGACTTCACAGGATTACATTaaCCTTTCTTTGAAGTGAATCATTTTATAGGAAGCCATTAGATGCTTAGGATAATCGAGGTAGAAAATGTGAGTCTTTGCTGTCAGTCTGGACTATGTAACATTAGGGCTCTCATAACTTGTTTCTTTCATTTTTCTTTTCTTTTCTTCTTTTAAAAGAATTACCAGGGCAAGCACACGTTGAATACAGCGGGGATTTCTTCTTCACAAGCTCCCAATCCAGTTGATGACGAGTCATGGGATgccaccaacttcagcctgctcaagcaggccggagatgtggaagagaaccccggccctATGgctccaaagaagaagcgtaaggtatccaatttactgaccgtacaccaaaatttgcctgcattaccggtcgatgcaacgagtgatgaggttcgcaagaacctgatggacatgttcagggatcgccaggcgttttctgagcatacctggaaaatgcttctgtccgtttgccggtcgtgggcggcatggtgcaagttgaataaccggaaatggtttcccgcagaacctgaagatgttcgcgattatcttctatatcttcaggcgcgcggtctggcagtaaaaactatccagcaacatttgggccagctaaacatgcttcatcgtcggtccgggctgccacgaccaagtgacagcaatgctgtttcactggttatgcggcggatccgaaaagaaaacgttgatgccggtgaacgtgcaaaacaggctctagcgttcgaacgcactgatttcgaccaggttcgttcactcatggaaaatagcgatcgctgccaggatatacgtaatctggcatttctggggattgcttataacaccctgttacgtatagccgaaattgccaggatcagggttaaagatatctcacgtactgacggtgggagaatgttaatccatattggcagaacgaaaacgctggttagcaccgcaggtgtagagaaggcacttagcctgggggtaactaaactggtcgagcgatggatttccgtctctggtgtagctgatgatccgaataactacctgttttgccgggtcagaaaaaatggtgttgccgcgccatctgccaccagccagctatcaactcgcgccctggaagggatttttgaagcaactcatcgattgatttacggcgctaaggatgactctggtcagagatacctggcctggtctggacacagtgcccgtgtcggagccgcgcgagatatggcccgcgctggagtttcaataccggagatcatgcaagctggtggctggaccaatgtaaatattgtcatgaactatatccgtaacctggatagtgaaacaggggcaatggtgcgcctgctggaagatggcgattgaAGCAAACGAACATACTTCAAGTCTGATAGTTTTGATGCAGAGAAGAAAGAGTTTTTATTTTTAAAATTTTAACAGAAGTGTGAAACCTGATATTCTTATATCTCCTGTTCTTCTGTTCTTCCTCCCAACCTTTAAAAAATAGCCAGCTTCATTGATTAGTTATGTGAAATGCTGACGTTACAACACTGCAGTTACTGATACAAATGGTGTTAGCTGGAAATATTAAAGCATTCTTATATGTTTTGCTTATTTCTAGTATATTCTTCAGAAAGTTAAGACATGTTTCATGTCCAAGTGCTTAAGTCTTAGTATAGTGTTTATGATCCTATAAAACAAGCAATAGGATGTGATGTGCTTTTGTTTAATTATTGGGTCTAATTTCTACTTGATCCTTTAAAAGAATAGTGTATCAGTACAATGTAATAACATGATTTTCATGAAACAGTAGAGACTGAAGCCTTTCAAAGTTATTTGATTTTTAGATCATCAGACATGTAATGAAAATGGTTCAGTTTGCAATGTGAGCTTTGTACTTGGTGGTATGACAAATGTTTGCTTTTATAATATACAGATTTTCCTTGCAAATAAAAGATGAAACACATTTttCCCTAAGTTTTCCAACAGCATTTTTCATTTTTCTTGTAGCACATTACAGTTCCTACCTTTCATATTTGGGTATTTTATATCTTGTGAGATCAGTATGGTGAGCAAG

*Icos* ss donor (arm: underline)

CACCGAGCCCTACCACATTTCCCCAGAGGGCACGTCTTTGTTCCTTAGTATAAATGATGGCAAATCAACACGCGTTTATCTGTTTAaaATCTGCGTCCCCCACCCCCATCCCCAGTGTGGAAgtcagcAAAGCGTGGAGACTATCTGAGTCATATTATCTAAACATGTTACTTGATACATAACATTTTATATTCTGTCTATCACATGAAAAGCCCATTTCTCATTAGGTGTATTCTAGTGCATAGTTTTCTCTTGTCACATTCTGGTTTTGTTTGCTTGGCTATAAGATCTTTCATTTTGGTATACATAGGAGTGGCTTTATAAAAAGAAATCACTTAAGTTCTCACTTAAAAATAATTATTATGGCAATGTGTTCACAACATAACACACTTGCTTTGTTCTCTTTTTTCATCCAGGAGAAATCAATGGCAGCGCCAATTATGAAATGTTTATATTTCATAACGGGGGCGTCCAAATTCTGTGTAAGTATCCCGACATCGTACAACAGTTTAAGATGCAGCTCTTGAAGGGCGGGCAGATATTGTGCGACCTGACCAAAACCAAAGGCAGCGGCAACACCGTTTCCATAAAGAGTCTTAAATTTTGCCACTCACAGCTCAGCAACAATTCTGTGAGTTTTTTTCTCTACAACTTGGACCATTCCCATGCAAACTATTACTTCTGTAACCTTTCAATTTTTGATCCTCCCCCTTTCAAAGTGACACTTACAGGAGGATATTTGCATATTTATGGTAAGGCATTGGCTGCTTTTATCATCGTGCCTTAAAAGTATATGCTCATCTCCAAGACTTCTCaaACCAAGGGAAATACACCTCTCTCTCTCTCTCTCTCTGTGTGTGTGTGTGTGTGTTGTGTTGAAATTCCTTTAGAAGTAGTTGACAGCAGCTCATTTTacATGAAATATTCATGAAATGATATGTCGTTAATATTAGTTGATATTTATTTGGAATAAGTGATTTATTTACATACACTGTTAAGTTAGAATTAGGGCACAGTCTTTCGAAATCTTTCATGACTCAGTGGCTATTCCGT

**Supplementary Table S2.** Primer sequences used in this study (5’-3’)

Knock-out identification primer of cell line

UPF1-KO-F TGAAGACCACATTTAGGCAACG

UPF1-KO-R ACCAGCACCCACCACAGAGC

SHROOM1-KO-F CACCCAGCCTCAGCACTCAT

SHROOM1-KO-R GGTAGGTAGGAAGCCCTTTCATTT

Gene editing validation primer

SLC36A3-cr1-F AGAAGAGGGCTCTTTACCTGTTGTA

SLC36A3-cr1-R TGTTAGTCAGGTTGGTCTTGAACTC

SLC36A3-cr2-F TTTTGCGAGCAGATTGAA

SLC36A3-cr2-R ATGCCAGTCCCATTCTTT

RABGAP1-cr1-F TGTATCAAAAGAAATGCTAATGATT

RABGAP1-cr1-R TGGTGGATGAAGCGGAGA

RABGAP1-cr2-F AATCTTTTGTAGGGTTAGAAGTGAC

RABGAP1-cr2-R TTTGGGACATGGGCTGTA

SHROOM1-cr1-F CCCTCTGTGCCTGTTATGT

SHROOM1-cr1-R GCAGAGTTTGAGAAGTGGG

SHROOM1-cr2-F GCAGCCTTTACCCTGAGAC

SHROOM1-cr2-R GAACAGGAGGGATGGTAAACT

SIRPB1-cr12-F GCAGTAGCGTCATCAGCA

SIRPB1-cr12-R AGGTGGGAAGGAAGCATT

UPF1-cr1-F TGACCCTGTTCCTTTACGCC

UPF1-cr1-R GGAAAGGGACTGAGGGAAGT

UPF1-cr2-F TCTGCTAATGGACCGTGAA

UPF1-cr2-R AAGCCGAGGAGGAAGACG

Knock-in identification primer of HDR assay

FBL-KI-F GACACACTAGAACCATAGCACTGAG

FBL-KI-R CCACCCAAGAACCGAAATCC

LMNA-KI-F AACGAATGCTTGGCTTTGA

LMNA-KI-R CCTGCGTTCTCCGTTTCC

Actb-KI-F CCGTAAAGACCTCTATGCC

Actb-KI-R CCTTCACCGTTCCAGTTT

Knock-in identification primer of mouse embryo injection

Ddx4-5’-F TTGCTGGTATTTGTTGGGATC

Ddx4-5’-R CCGCCGCATAACCAGTGAA

Ddx4-3’-F GTTAGCACCGCAGGTGTAGAG

Ddx4-3’-R GAGGAAACTGAAATACCATG

ICOS-5’-F GACTCATGCTGTGGTTTCAGG

ICOS-5’-R GCTGAGCTGTGAGTGGCAA

ICOS-3’-F CCAAATTCTGTGTAAGTATCCCGAC

ICOS-3’-R GGCTCACCTCATTCTCCAAGC

**Supplementary** **Table S3.** siRNA, sgRNA target and crRNA target sequences used in this study (5’-3’)

siRNA sequences

*SLC36A3*-Homo-1742: GGUGUAUAUUUGGGACAUATT

UAUGUCCCAAAUAUACACCTT

*SLC36A3*-Homo-1516: GGUUGUACCAGUCAGUCAATT

UUGACUGACUGGUACAACCTT

*SLC36A3*-Homo-540: GGACUAUCGAUGAUGCAAATT

UUUGCAUCAUCGAUAGUCCTT

*SLC36A3*-Homo-685: GCAUGGUCAUCCUGUUGAATT

UUCAACAGGAUGACCAUGCTT

*RABGAP1*-Homo-425: CCGCUUCAUCCACCAUUAATT

UUAAUGGUGGAUGAAGCGGTT

*RABGAP1*-Homo-1088: GCCAAGGAAUUGAUAAGAATT

UUCUUAUCAAUUCCUUGGCTT

*RABGAP1*-Homo-1936 GCUAUCACCCGGGAUAUUATT

UAAUAUCCCGGGUGAUAGCTT

*SHROOM1*-Homo-1576 (*SHROOM1’*) CCAAACUUUCCAGGUUCUUTT

AAGAACCUGGAAAGUUUGGTT

*SHROOM1*-Homo-1938 GGUAUUUCCAGGCCCACAATT

UUGUGGGCCUGGAAAUACCTT

*SHROOM1*-Homo-1977 GCAAAUGAUAACAUCCCAATT

UUGGGAUGUUAUCAUUUGCTT

*TNRC6B*-Homo-5262 GCACUGCCCUGAUCCGAUATT

UAUCGGAUCAGGGCAGUGCTT

*SIX1*-Homo-836 CCAACAAGCAGAACCAACUTT

AGUUGGUUCUGCUUGUUGGTT

*CSDE1*-Homo-537 CCACAACAAUGGACAUAAUTT

AUUAUGUCCAUUGUUGUGGTT

*SIRPB1*-Homo-990 CUUCGACCCUCAUAGAGAATT

UUCUCUAUGAGGGUCGAAGTT

*HDGF*-Homo-158 CAGACGUCCACACUUAACUTT

AGUUAAGUGUGGACGUCUGTT

*UPF1*-Homo-1169 CCUACCAGUACCAGAACAUTT

AUGUUCUGGUACUGGUAGGTT

*Shroom1*-Mus-1889 GCAUCGGAGGAAGGGUCUUTT

AAGACCCUUCCUCCGAUGCTT

*Shroom1*-Mus-1394 (*shroom1’*) GCUGGUCAGAUUGCAGUUUTT

AAACUGCAAUCUGACCAGCTT

sgRNA target sequence (PAM: underline)

*BFP* GCTGAAGCACTGCACGCCATGGG

*FBL* CTCTCACAGGCCACCCCCCAAGG

*LMNA*  CCATGGAGACCCCGTCCCAGCGG

*Actb*  AGTCCGCCTAGAAGCACTTGCGG

*SHROOM1-1*  TCTGTCCATGCGCGCGGACTCGG

*SHROOM1-2*  GCGCTCCGACTCCGACTGGCGGG

*Ddx4-1* GACTTCACAGGATTACATTGG

*Ddx4-2* GCTGTTGGAAAACTTAGGGGG

*ICOS-1* TAGTCTCCACGCTTTTACAGG

*ICOS-2* GTTGACAGCAGCTCATTTTGG

crRNA target sequence (PAM: underline)

*UPF1-1*  TTTCATGTGCTCAGACTCAAGATAACAT

*UPF1-2*  TTTACCTCAAGACATGCGGCTCATGCAG

*SLC36A3-1* TTTAAGCCACTACAGACCGACACCTGG

*SLC36A3-2* TTTCCTTCCCAGGTACACTGTCAGCTT

*RABGAP1-1* TTTGTGACCCTTAGATTGTAGGGAATG

RABGAP1-2 TTTCCTCATTTATTAAATGAGGTGGCT

*SHROOM1-1* TTTCCCTCTCAGAACCTCATTGTCCTT

*SHROOM1-2*  TTTATCAGGGAAAGATCACATGGAGAA

*SIRPB1-1*  TTTCACAATCCAGAAGCAGGTTACTGC

*SIRPB1-2*  TTTGGACAGAGCAGGCTCCTAAGGTCT

*AAVS1-1*  TTTCATTTGGGCAGCTCCCCTACCCCC

*AAVS1-2* TTTCTGTCTGCAGCTTGTGGCCTGGGT
